# Summative assessments are more powerful drivers of student learning than resource intensive teaching formats

**DOI:** 10.1186/1741-7015-11-61

**Published:** 2013-03-05

**Authors:** Tobias Raupach, Jamie Brown, Sven Anders, Gerd Hasenfuss, Sigrid Harendza

**Affiliations:** 1 Department of Cardiology and Pneumology, University Hospital Göttingen, Robert-Koch-Straße 40, Göttingen, D-37075, Germany; 2 Health Behaviour Research Centre, University College London, 1-19 Torrington Place, London, WC1E 7HB, UK; 3 Department of Legal Medicine, University Medical Centre Hamburg-Eppendorf, Butenfeld 34, Hamburg, D-22529, Germany; 4 Department of Internal Medicine, University Medical Centre Hamburg-Eppendorf, Martinistraße 52, Hamburg, D-20246, Germany

**Keywords:** assessment, electrocardiogram, medical education, teaching

## Abstract

**Background:**

Electrocardiogram (ECG) interpretation is a core clinical skill that needs to be acquired during undergraduate medical education. Intensive teaching is generally assumed to produce more favorable learning outcomes, but recent research suggests that examinations are more powerful drivers of student learning than instructional format. This study assessed the differential contribution of teaching format and examination consequences to learning outcome regarding ECG interpretation skills in undergraduate medical students.

**Methods:**

A total of 534 fourth-year medical students participated in a six-group (two sets of three), partially randomized trial. Students received three levels of teaching intensity: self-directed learning (two groups), lectures (two groups) or small-group peer teaching facilitated by more advanced students (two groups). One of the two groups on each level of teaching intensity was assessed in a formative, the other in a summative written ECG examination, which provided a maximum of 1% credit points of the total curriculum. The formative examination provided individual feedback without credit points. Main outcome was the correct identification of ≥3 out of 5 diagnoses in original ECG tracings. Secondary outcome measures were time spent on independent study and use of additional study material.

**Results:**

Compared with formative assessments, summative assessments increased the odds of correctly identifying at least three out of five ECG diagnoses (OR 5.14; 95% CI 3.26 to 8.09), of spending at least 2 h/week extra on ECG self-study (OR 4.02; 95% CI 2.65 to 6.12) and of using additional learning material (OR 2.86; 95% CI 1.92 to 4.24). Lectures and peer teaching were associated with increased learning effort only, but did not augment examination performance.

**Conclusions:**

Medical educators need to be aware of the paramount role of summative assessments in promoting student learning. Consequently, examinations within medical schools need to be closely matched to the desired learning outcomes. Shifting resources from implementing innovative and costly teaching formats to designing more high-quality summative examinations warrants further investigation.

## Background

Most medical school curricula have adopted innovative teaching methods such as problem-based learning [1] and student-led peer teaching [2]. According to their theoretical underpinnings [3], these are thought to enhance student learning, performance in examinations and, eventually, clinical competence. One could therefore expect these methods to produce a substantially greater performance gain than traditional teaching methods (that is, lectures) or even self-directed learning in the absence of formal teaching. However, while numerous studies have provided evidence of non-inferiority of innovative teaching methods when compared to traditional instructional formats [4,5], the way student performance was assessed has not been taken into account in these studies. Research suggests that assessments may be more important for student learning than the choice of instructional format.

Three decades ago, Newble and Jaeger observed a significant effect of assessments on the learning behavior of medical students [6]. Since then, the axiom 'assessment drives learning' [7] has been widely accepted as a fundamental rule of medical education, even to the extent of characterizing assessments as 'educational tools' [8]. This wide acceptance is despite a substantial lack of high-quality research into the nature of the association between assessment and learning [9]. For example, the extent to which examinations impact on student learning behavior may be crucially dependent on their consequences: Formative (that is, feedback-generating [10]) assessments may generate a smaller incentive to learn than summative (that is, graded [11]) assessments as students can potentially fail the latter. So far, no study has directly compared the differential contribution of teaching intensity and assessment consequences to learning outcome in medical education. Given the substantial resource requirements of some innovative teaching methods, knowledge of their impact on student learning relative to the impact of assessments is also important from a cost-effectiveness point of view.

Swift identification of patients with ST segment elevation myocardial infarction is crucial to initiating treatment without delay in order to keep morbidity and mortality to a minimum [12,13]. In the interest of patient safety, physicians of all specialties must be familiar with the basic principles of electrocardiogram (ECG) interpretation as diagnostic errors based on ECG readings can result in adverse patient outcome [14]. However, there have been numerous reports of insufficient ECG interpretation skills in physicians [15]. For example, less than half of doctors surveyed in a recent study were able to correctly measure the QT interval [16], and one in five family practice residents included in one study failed to diagnose an acute myocardial infarction from an ECG tracing [17]. In 2011, 60% of a cohort of 637 junior doctors in Germany reported feeling inadequately prepared for postgraduate training, and self-assessed deficits in ECG interpretation were independently associated with this belief [18]. Given the relevance of basic ECG interpretation skills in all medical specialties, these skills must be acquired effectively during undergraduate medical education. However, there remains considerable uncertainty regarding the ideal teaching format to achieve this goal [19].

The aim of the present study was to examine the effect of three teaching formats and two different consequences of assessments (formative vs summative) on student performance in a written test of ECG interpretation skills. We hypothesized assessment consequences to have a greater impact on student learning behavior and learning outcome than teaching format.

## Methods

### Study design

**Figure 1:**
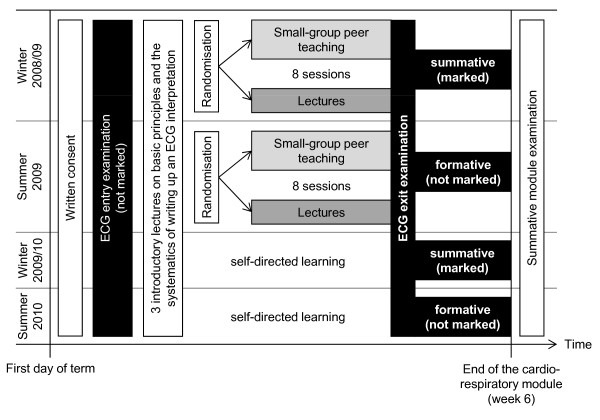
**Schematic diagram of the study design**. The six study groups differed with regards to assessment consequences (summative/formative) and teaching format (self-directed learning/lectures/small-group peer teaching).

We carried out a six-group (two sets of three), partially randomized and single-blinded trial among four consecutive cohorts of fourth-year medical students enrolled in a 6-week cardiorespiratory module at Göttingen Medical School (Figure 1). At the beginning of the module, all students were provided with a 40-page guide to ECG interpretation and were offered 3 introductory lectures on the basic principles of ECG interpretation. Specific diagnoses were not discussed in these lectures. Students in the first two cohorts (winter 2008/2009 and summer 2009) were stratified by sex and previous end-of-module examination scores. Within these groups, students were then randomized to eight sessions of large-group teaching (traditional lectures) or small-group teaching led by more advanced medical students (peer teaching). Students in the third and fourth cohort (winter 2009/2010 and summer 2010) did not receive any additional formal teaching. All students took a formative ECG entry examination; the consequences of the exit examination at the end of the module differed between groups: The test was summative in the first and the third cohort and formative in the second and fourth cohort.

### Teaching methods

Three levels of teaching intensity were used in this study. The lowest level (referred to as 'self-directed learning' (SDL)) did not involve any formal teaching apart from three introductory lectures on basic principles of ECG interpretation. However, students were encouraged to self-study the 40-page guide containing examples of typical ECG tracings. The second level of teaching intensity (referred to as 'lectures') consisted of eight 45-minute large-group sessions during which an expert electrocardiographer discussed a number of ECG tracings from the ECG interpretation guide. The highest level of teaching intensity, (referred to as 'peer teaching'), consisted of eight 45-minute small-group sessions facilitated by near-peers, that is, medical students in their fifth year who had been specifically trained as student teachers according to current recommendations [20]. During small-group sessions, eight to nine medical students discussed the same ECG tracings that were presented in lectures. In contrast to the expert electrocardiographers facilitating lectures, peer teachers were not supposed to answer questions but were trained to stimulate group discussion and help students to find solutions to their problems collectively. In order to avoid contamination between lectures and small-group sessions, teaching sessions were run in parallel, and students were unable to switch group assignments.

### ECG examination consequences

Two types of examination consequences were used in the study; tests were either 'summative' or 'formative'. The summative ECG exit examinations generated credit points relevant for students' overall marks at the end of undergraduate medical education. At the institution in which the research was conducted, a maximum of 100 credit points could be scored in each of 33 specialties, adding up to a maximum score of 3,300 points at the end of the clinical curriculum. Raw points scored in summative ECG examinations were converted into credit points with a maximum of 7 points per ECG tracing, thus providing students with a chance of scoring up to 35 credit points in the exit examination. This equaled 1% of all available credit points, which was deemed an adequate incentive for students to engage in learning how to interpret an ECG. The formative ECG exit examinations did not generate any credit points for students (the 35 points available to students with a summative ECG examination were assigned to other examinations within the curriculum in cohorts with a formative ECG examination). Individual feedback was provided in terms of the total score achieved by each student, but no further discussion of results was offered as this would have interfered with the study design (identical ECG tracings were used in all cohorts).

### Assessment tools

Students were asked to complete two written tests of ECG interpretation: one at the beginning (entry examination) and one at the end (exit examination) of the module. Only unambiguous tracings of ECGs with medically important findings selected by electrocardiographers were used for assessments. Expert electrocardiographers produced correct interpretations of these tracings, against which student interpretations were compared. The entry examination contained three ECG tracings (normal ECG, acute myocardial infarction, and right bundle branch block), and the exit examination contained five different ECG tracings (acute myocardial infarction, AV conduction block II°, atrial fibrillation, left ventricular hypertrophy, and QT prolongation). None of these tracings were available to students or teachers (lecturers/near-peers), and ECGs used for assessments were not included in the 40-page guide. Students were asked to provide a full written interpretation of rhythm, rate, axis, conduction times, signs of hypertrophy and ST segment abnormalities. We used a validated scoring system [21] yielding a maximum score of ten points per ECG tracing. Two raters blinded to teaching intensity independently scored examinations, and inter-rater agreement was high (weighted kappa 0.95 for the exit examination).

All students took a summative end-of-module examination that is part of the official curriculum at the institution where the study was performed. This examination consisted of 69 multiple choice questions on the diagnosis and treatment of cardiorespiratory disease; the Cronbach α of the exam was >0.75 in all cohorts. The end-of-module examination was completely unrelated to the study; the topic of ECG interpretation was not included in that examination. However, we obtained student consent to use percentage scores achieved in this examination as indicators of student performance levels and include them in subsequent analyses (see below).

### Questionnaires

All study participants were asked to complete an entry questionnaire on the first day of the six-week module. In addition to collecting information on age and sex, the questionnaire required students to self-rate seven statements on six-point scales. These were related to learning style, motivation to learn how to interpret an ECG, and expectations towards the module. The wording of these statements is provided in Table 1. As part of the ECG exit examination, students were asked to indicate how many hours per week they had spent on voluntary ECG self-study (in addition to scheduled teaching sessions) and whether they had used additional ECG learning material during the module.

**Table 1:** Student characteristics, self-ratings and scores in the electrocardiogram (ECG) entry examination as well as the summative end-of-module examination the six study groups.

Term	Winter 2008/2009	Winter 2008/2009	Summer 2009	Summer 2009	Winter 2009/2010	Summer 2010	ANOVA/χ^2 ^test
Number of students	82	80	81	77	148	66	
Teaching format	Lectures	PT	Lectures	PT	SDL	SDL	
Assessment consequences	Summative	Summative	Formative	Formative	Summative	Formative	
Age, years	23.9 (2.4) ± 0.5	24.1 (2.7) ± 0.6	24.0 (1.8) ± 0.4	24.1 (2.4) ± 0.5	24.5 (2.6) ± 0.4	24.6 (2.7) ± 0.7	F = 0.920; *P *= 0.479
Percentage score achieved in the ECG entry examination	26.7 (14.0) ± 3.1	26.8 (13.6) ± 3.0	20.0 (12.8) ± 2.9	20.8 (12.6) ± 2.9	25.2 (14.3) ± 2.4	24.0 (13.8) ± 3.4	F = 3.747; *P *= 0.002
Percentage score in the summative end-of-module module examination	80.3 (8.2) ± 1.8	79.6 (8.6) ± 1.9	74.6 (8.5) ± 1.9	76.6 (7.3) ± 1.7	79.8 (9.4) ± 1.6	77.2 (9.9) ± 2.4	F = 5.794; *P *<0.001
Female sex, % (n)	59.8 (49)	58.8 (47)	58.0 (47)	57.1 (44)	52.7 (78)	63.6 (42)	χ^2 ^= 2.646; *P *= 0.754
'I need some external pressure in order to be motivated to learn', % (n) agreement	45.1 (37)	38.8 (31)	44.4 (36)	40.3 (31)	31.8 (47)	33.3 (22)	χ^2 ^= 6.415; *P *= 0.268
'Preferably, I learn those things that will be tested in exams', % (n) agreement	62.2 (51)	51.3 (41)	64.2 (52)	54.5 (42)	52.7 (78)	48.5 (32)	χ^2 ^= 6.364; *P *= 0.272
'In my view, the electrocardiogram (ECG) as an important diagnostic tool', % (n) agreement	98.8 (81)	98.8 (79)	95.1 (77)	96.1 (74)	98.6 (146)	93.9 (62)	χ^2 ^= 6.857; *P *= 0.231
'I am looking forward to learning something about ECG interpretation in this module', % (n) agreement	93.9 (77)	92.5 (74)	87.7 (71)	89.6 (69)	87.2 (129)	87.9 (58)	χ^2 ^= 3.801; *P *= 0.578
'I have read a book on ECG interpretation before', % (n) agreement	32.9 (27)	25.0 (20)	12.3 (10)	15.6 (12)	20.3 (30)	13.6 (9)	χ^2 ^= 15.251; *P *= 0.009
'I have already learned some bits and pieces about the ECG prior to this module', % (n) agreement	8.5 (7)	7.5 (6)	2.5 (2)	7.8 (6)	3.4 (5)	4.5 (3)	χ^2 ^= 5.741; *P *= 0.332
'I expect to be taught all the relevant facts and skills about ECG interpretation during the teaching sessions of the cardiovascular module', % (n) agreement	74.4 (61)	85.0 (68)	84.0 (68)	90.9 (70)	88.5 (131)	89.4 (59)	χ^2 ^= 12.098; *P *= 0.033

Data are presented as mean (SD) ± standard error or % (n) as appropriate. ANOVA = analysis of variance; PT = peer teaching; SDL = self-directed learning.

### Student enrolment, data collection and analysis

At 4 weeks before the start of the module, medical students were informed about the study by email. On the first day of the module, all students were asked whether they would provide written consent to participate in the study, and consenting students completed the entry questionnaire and the ECG entry examination. The ECG exit examination was scheduled during the final week of the module, 3 days before the summative end-of-module examination. In order to avoid contamination between student cohorts, all test materials were collected after each assessment.

Descriptive analyses of demographic variables, student self-ratings and scores in all ECG examinations as well as the summative end-of-module examination were conducted separately for each of the six study groups, and differences between groups were assessed by χ^2 ^tests (dichotomous variables) and analysis of variance (ANOVA; continuous variables). Student ratings on six-point scales were dichotomized by collapsing the two most positive options and the remaining four options into positive and neutral/negative categories, respectively. The primary outcome for this study was the correct identification in the exit examination of at least three out of the five diagnoses listed above. Student self-reports of having spent more than 2 h/week on independent ECG self-study and of having used additional ECG learning material during the module were used as secondary outcomes. Multivariate regression analyses adusting for sex, age, performance levels, and initial self-ratings were used to predict primary and secondary outcomes. Formative examinations and the lowest level of teaching intensity (self-directed learning) were used as reference for these analyses, and results are given as odds ratios and 95% confidence intervals. The interaction between teaching intensity and assessment consequences was tested by adding interaction terms to the models. To validate the primary measure of student performance, we also conducted a sensitivity analysis in which we used an ANOVA to examine the effects of teaching format and assessment consequences on the percentage score in the ECG exit exam. Statistical analysis was performed using SPSS 19.0 (SPSS Inc., Chicago, IL, USA). Data are presented as mean ± standard deviation or percentages (n), as appropriate. Significance levels were set to *P *<0.05. This study was approved by the local Ethics Committee (Ethik-Kommission der Medizinischen Fakultät der Georg-August-Universität Göttingen; application numbers 23/2/09, 18/8/09 and 1/3/10).

## Results

Of the 565 students eligible for study participation, only 1 failed to provide written consent. A total of 30 students were excluded due to missing data in the entry questionnaire or the ECG exit examination. Complete data were therefore available for 534 students. The mean age of study participants was 24.2 ± 2.5 years, and 57.5% (n = 307) were women. One in five (20.2%, n = 108) students entering the fourth year of undergraduate education indicated they had read a book on ECG interpretation before the module, and 5.4% (n = 29) stated they had engaged in more detailed voluntary learning activities regarding ECG interpretation in the past. The majority of students agreed that the ECG was an important diagnostic tool (97.2%, n = 519) and that they looked forward to learning how to read an ECG during the module (89.5%, n = 478). At the same time, 85.6% (n = 457) expected to be taught all relevant facts and skills during face-to-face teaching sessions of the module. With regards to the impact of examinations, only 38.2% of students (n = 204) stated that they needed some external pressure in order to be motivated to learn, and 55.4% of students (n = 296) admitted to preferentially learning content that they knew would be tested in examinations. Student characteristics by study group are provided in Table 1. There were significant differences between the six cohorts in performance in the ECG entry examination and the summative end-of-module examination as well as in the percentage of students reporting to have read an ECG book before the module and expecting to be taught all relevant aspects of ECG interpretation during face-to-face sessions. These differences were accounted for in the adjusted multivariate model.

**Figure 2:**
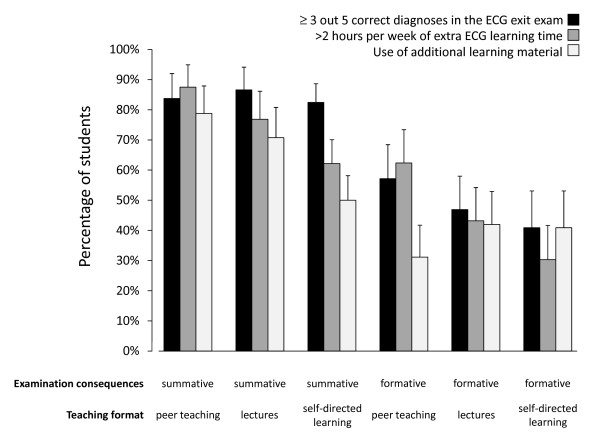
**Descriptive analysis of primary and secondary outcomes**. The figure shows percentages of students correctly identifying at least three out of five diagnoses in the electrocardiogram (ECG) exit examination (black columns), self-reporting to have spent more than 2 h/week on independent ECG self-study (dark gray columns) and of having used additional ECG learning material during the module (light gray columns) by study group. Error bars indicate 95% confidence intervals of prevalence estimates.

Overall, 69.1% (n = 369) of students correctly identified at least three out of five diagnoses in the ECG exit examination, 61.4% (n = 328) self-reported having spent more than 2 h/week on independent ECG self-study, and 52.4% (n = 280) indicated having used additional ECG learning material during the module. Figure 2 displays primary and secondary outcomes as a function of study group. The percentage of students correctly identifying at least three out of five diagnoses was above 80% in all groups with summative examinations and below 60% in all groups with formative examinations.

Results of the multivariate logistic regression analysis adjusting for all baseline variables and student performance in the summative end-of-module examination are presented in Table 2. The only significant predictor of the primary outcome was examination consequences: those allocated to a summative examination had more than five times the odds of being able to correctly identify three out of five diagnoses than those allocated to a formative examination.

**Table 2:** Predictors of primary and secondary outcomes in a multivariate regression model adjusting for sex, age, performance level, and initial self-ratings.

Predictors		Adjusted odds ratios (95% confidence interval)		
		
		Primary outcome: ≥3 out of 5 correct diagnoses	Secondary outcome: >2 h/week of extra ECG learning time	Secondary outcome: use of additional learning material
Examination consequences	Formative	1.00 (reference)	1.00 (reference)	1.00 (reference)
	Summative	**5.14 (3.26 to 8.09)**	**4.02 (2.65 to 6.12)**	**2.86 (1.92 to 4.24)**

Teaching format	Self-directed learning only	1.00 (reference)	1.00 (reference)	1.00 (reference)
	Lectures	1.50 (0.87 to 2.56)	**2.14 (1.33 to 3.45)**	**1.94 (1.22 to 3.01)**
	Small-group peer teaching	1.62 (0.95 to 2.76)	**4.42 (2.64 to 7.38)**	**1.81 (1.15 to 2.87)**

Significant results are displayed in bold letters. ECG = electrocardiogram.

Examination consequences also predicted the secondary outcomes of student learning behavior with summative examinations increasing the odds of spending more than 2 h/week on voluntary ECG self-study by four and the odds of using additional learning material by three. Teaching intensity predicted learning behavior but not examination performance: compared with students who did not receive any formal teaching, students randomized to receiving eight lectures were more likely to spend more time on ECG self-study and use additional learning materials. Similarly, peer teaching significantly increased the odds of spending more time on self-study and using additional learning material. Among students receiving peer teaching, the odds of spending more than 2 h/week on independent ECG self-study were more than four times those in the self-directed learners. In contrast, students receiving lectures had only 1.8 times the odds of spending more than 2 h/week compared to self-directed learners.

Possible effects of an interaction between examination consequences and teaching intensity were assessed by including interaction terms in the models. The odds ratio of the effects of summative versus formative examinations by the effects of different levels of teaching (OR*_int_*) did not yield any significant results for the primary outcome (OR*_int _*for lectures vs SDL: 0.69; 95% CI 0.23 to 2.06; OR*_int _*for peer teaching vs SDL: 0.44; 95% CI 0.15 to 1.28) and the secondary outcome 'learning time' (OR*_int _*for lectures vs SDL: 1.11; 95% CI 0.43 to 2.86; OR*_int _*for peer teaching vs SDL: 1.06; 95% CI 0.37 to 2.99). Regarding the other secondary outcome (use of additional learning material), both effects were similar when comparing lectures to SDL (OR*_int _*1.87; 95% CI 0.75 to 0.64) but the effect of examination consequences was significantly stronger in students receiving peer teaching than in students engaging in self-directed learning (OR*_int _*5.38; 95% CI 2.06 to 14.09).

In a sensitivity analysis using a continuous primary outcome measure, an ANOVA assessing the effects of examination consequences and teaching intensity on the actual percentage score achieved in the ECG exit exam and controlling for performance in the ECG entry exam yielded a small but significant effect of teaching format (η^2^_p _= 0.012; *P *= 0.047) and a much larger effect of examination consequences (η^2^_p _= 0.328; *P *<0.001). There was no interaction between examination consequences and teaching intensity (η^2^_p _= 0.005; *P *= 0.272).

## Discussion

ECG interpretation is a core clinical skill that needs to be acquired during undergraduate medical education [13]. This is the first study to compare the relative impact of different levels of teaching and different consequences of examinations on student performance of a clinical skill. Confirming our hypothesis, we found a strong association between summative examinations and better performance in the ECG exit examination while teaching intensity did not predict student performance.

### Comparison with other studies

In 2005, a survey of Clerkship Directors in Internal Medicine in the US revealed that the predominant instructional format for ECG interpretation was large-group teaching with 75% of medical schools offering lectures to teach ECG reading skills [19]. A number of studies have assessed the effect of different instructional formats on student ECG interpretation skills [22,23]. Comparability of these studies is limited as different methods were used to measure student performance (for example, multiple choice tests, open questions), and most studies failed to report whether examinations were formative or summative. The available literature suggests that large-group teaching is more effective than no teaching [24]. More recently, Mahler *et al*. reported that self-directed learning was inferior to lectures and workshops in promoting ECG interpretation skills [25]. This resonates with our current findings, but that study did not allow any conclusions to be drawn regarding the effect of examination consequences on student performance. Moreover, it has not been assessed whether examination consequences have a moderating effect on the effectiveness of different levels of teaching intensity. To that end, we assessed the interaction between examination consequences and teaching intensity with regard to their effects on student performance and learning behavior and found no significant interaction for performance in the ECG exit exam and student learning time. In accordance with the unadjusted data presented in Figure 2, we found a significantly greater effect of examination consequences on the use of additional learning material in the context of peer teaching than in the context of SDL. It might be hypothesized that students in the SDL condition might not have been as motivated to consult additional learning material even in the face of a summative exam as students experiencing the benefits of peer teaching. This hypothesis should be tested in future studies. However, the overall effect of examination consequences appeared to be independent of the effect of teaching intensity on student performance.

Our study provides some evidence that teaching format does impact on learning behavior. As expected from underlying theory [2], small-group peer teaching was more effective in stimulating self-directed learning than lectures, and this finding is important with regard to preparing undergraduate medical students for lifelong learning in clinical medicine. In fact, an ANOVA using the percentage score of points achieved in the ECG exit exam (rather than the percentage of students correctly identifying ≥3 out of 5 diagnoses) as the dependent variable showed that higher teaching intensity was significantly associated with better exam performance but that effect was much smaller than the effect of examination consequences on percentage score.

Taken together, our data suggest that identifying the 'ideal' teaching format might be futile if learning is not adequately incentivized by an adequate summative assessment that is matched to the learning objective.

Owing to the dominance of psychometric theory during the second half of the 20th century, great emphasis was put on the numerical aspects of assessments in medical education. In contrast to this, assessments are now perceived as being at the heart of the educational design [26]. In this regard, the paucity of research into the mechanisms by which assessments guide student learning is surprising [27], particularly in the light of the repeated calls for such research [9,26]. The fact that, in our present study, a summative assessment was the only significant predictor of student performance even after adjusting for motivation questions the general notion that medical students' motivation to learn is mainly driven by the aspiration of becoming a 'good doctor' [28]. It also contradicts the 'andragogy hypothesis' which states that adult learners are intrinsically motivated to learn because they acknowledge the relevance of the content taught to the professional activity for which they are training [29]. While this hypothesis has already been challenged on theoretical grounds [30], we here provide data suggesting that summative examinations generate a strong extrinsic motivation to learn that may even override intrinsic motivation. Finally, it should be noted that medical students are a diverse population, and the impact of examination consequences and teaching format may vary greatly between individuals. This study was not designed to identify subgroups that benefit most from interactive teaching, but such research is clearly needed to help medical educators design curricula that are tailored to their students' needs. In addition, it would be interesting to assess how student experiences with different teaching formats gained in this study impact on subsequent learning behavior (that is, students in the SDL condition who scored highly in the ECG exit exam might feel more confident to engage in SDL activities and become less dependent on didactic teaching).

### Strengths and limitations of the study

The design of this study allowed the identification of predictors of student performance in a reliable test of ECG interpretation skills. Since production tests are regarded superior to recognition tests [11], we used a written examination format and did not provide predefined answers. We enrolled over 500 undergraduate medical students and obtained complete data for over 94% of eligible participants, thus rendering any selection bias unlikely. All differences in baseline performance levels between the six groups were adjusted for in the multivariate analysis. In order to allow comparisons across groups, identical ECG examinations were used in all groups. We took great care to collect all test materials after each examination, and the marginally weaker performance of the final cohort suggests that these students did not have access to any examination materials, thus rendering contamination bias unlikely.

The trial was only partially randomized as ethical reasons prohibited randomizing students of the same cohort to either summative or formative examinations; this would have disadvantaged students who would not have been able to score additional credit points in the ECG exit examination. As the reference conditions of SDL and a formative assessment were only used in the final cohort, we cannot entirely rule out a potential historical threat to validity as that cohort might have had different experiences than the other ones. However, as far as the baseline variables were concerned, there was no evidence of the final cohort being any different from the others.

Learning and performance in examinations have been shown to be case specific [31]. The sampling used for the primary outcome of this study may have been insufficient; however, including more ECG tracings in the exit examination would have increased the time required to complete the test, thereby increasing the risk of higher dropout rates in study groups with a formative examination. In addition, reanalyzing the data using raw point scores did not change the results, suggesting that the approach used in our analysis was valid. Our study was conducted at one German medical school, and we only assessed one learning objective. Future research needs to determine whether our findings generalize across cognitive, practical and affective learning objectives, medical curricula and countries. Finally, we did not assess long-term retention of ECG interpretation skills. Given that the impact of problem-based learning on retention might only become apparent after longer periods of time [32], future studies should investigate the effect of examination consequences and teaching format during undergraduate medical education on performance in residency. However, control of confounding is particularly challenging in this type of study.

## Conclusions

To the best of our knowledge, this study demonstrates for the first time that summative assessments drive student learning to a much greater extent than innovative instructional formats that were deliberately designed to enhance student learning. The most important consequence of this finding for medical education is that medical educators must be aware of the huge influence of assessments on student learning behavior. Examinations should therefore be designed with great care. Recognizing summative examinations as the main driving force of student learning also demands the prioritization of learning objectives, as the capacity for testing during medical education is limited. Medical schools should strive to agree upon a set of learning objectives that are considered crucial for every physician. Concentrating resources on the design and implementation of valid summative examinations may prove more cost effective in the long run than trying to identify the optimal teaching method for each learning objective.

## Abbreviations

ANOVA: analysis of variance; CI: confidence interval; ECG: electrocardiogram; OR*_int _*: odds ratio for interaction; PT: peer teaching; SDL: self-directed learning.

## Competing interests

The authors declare that they have no competing interests.

## Authors' contributions

TR conceived of the study, developed its design, was involved in data analysis and wrote the manuscript. JB was involved in data analysis and contributed to the Introduction and Discussion section. SA helped to design the study, provided advice on data presentation and commented on various versions of the manuscript. GH drafted the abstract, contributed to the discussion and provided comments on the manuscript. SH helped to design the study, identified relevant literature, contributed to the discussion and commented on various versions of the manuscript. All authors have approved the final version of this article.

## Authors' information

TR is a cardiologist who works in the Department of Cardiology and Pneumology at Göttingen University. He co-ordinates the department's teaching activities and has helped to develop the institution's curriculum. His current research focuses on curricular development, evaluation and assessment formats.

JB is a psychologist affiliated to the Institute of Epidemiology & Health at University College London. His main research focus is smoking cessation.

SA works as a consultant in the Department of Legal Medicine at Hamburg University, coordinating the department's teaching activities. He is involved in curricular development and has completed a 2-year study course of Medical Education. Main research areas are forensic pathology, clinical forensic medicine, and medical education.

GH is chief of the Department of Cardiology and Pneumology and chair of the Heart Centre at Göttingen University. His main research interests are molecular pathophysiology and the treatment of heart failure including cardiac stem cell biology. He is lecturing in the department's 6-week cardiorespiratory teaching module.

SH is assistant professor for internal medicine/nephrology and was vice-dean of education from 2006 to 2007 at the Medical Faculty of Hamburg University, Germany. She received an MME degree at Bern University, Switzerland, and the Ars legendi award 2006 for medical education. She teaches educational management in the German MME program.

## Pre-publication history

The pre-publication history for this paper can be accessed here:

http://www.biomedcentral.com/1741-7015/11/61/prepub
